# Antibacterial and antibiofilm activities of cinnamon essential oil nanoemulsion against multi-species oral biofilms

**DOI:** 10.1038/s41598-021-85375-3

**Published:** 2021-03-15

**Authors:** Yeo-Jin Jeong, Hee-Eun Kim, Su-Jin Han, Jun-Seon Choi

**Affiliations:** 1 Department of Health Science, Graduate School of Gachon University, Incheon, Republic of Korea; 2 Department of Dental Hygiene, College of Health Science, Gachon University, 191 Hambakmoero Yeonsu-gu, Incheon, 21936 Republic of Korea

**Keywords:** Diseases, Health care

## Abstract

Cinnamon essential oil (CEO) has antibacterial properties, but its ability to suppress the formation of multi-species oral biofilms has not been fully elucidated. This study evaluated the antibacterial and antibiofilm activities of cinnamon essential oil nanoemulsion (CEON) against oral biofilms formed using a microcosm biofilm model. The biofilms were formed on bovine enamel specimens over a 7-day period, during which all specimens were treated with one of three solutions: 5% CEON (n = 35), 0.5% cocamidopropyl betaine (n = 35), or 0.12% chlorhexidine gluconate (CHX; n = 35). Antibacterial and antibiofilm activities were determined by the red/green ratios (R/G values) of 7-day-old mature biofilms photographed with quantitative light-induced fluorescence-digital, the number of aciduric bacterial colony-forming units (CFUs) within each biofilm, and the absorbance of bacterial suspensions. One-way and repeated-measures analysis of variance were performed to compare differences among the three solutions. R/G values were lowest in the 0.12% CHX group, but not significantly differ from the 5% CEON group. The number of CFUs and absorbance were lowest in the 5% CEON group. This study showed that nanoemulsified CEO inhibited the maturation of multi-species oral biofilms and the growth of oral microorganisms in biofilms, including aciduric bacteria that cause dental caries.

## Introduction

Dental caries and periodontal diseases are common worldwide and represent the main causes of tooth loss^1^. Careful management of oral biofilms, which consist of complex microbial communities, is critical to prevent the onset and progression of oral diseases^2^. Compared with planktonic bacteria, microbial communities that have formed biofilms are more resistant to external stress (e.g., nutrient depletion or toxic substance exposure), due to the presence of bacterial extracellular polysaccharides^3,4^. Therefore, residual oral biofilms comprising pathogenic bacteria that have not been removed for extended periods of time may be increasingly hazardous to oral health^5^.

Although biofilm removal is considered important for maintaining oral health, the control of biofilms may be inadequate for many people^6^. Notably, although tooth brushing is considered the first-line oral hygiene care method, its biofilm-removal effect may be limited in certain patients or specific areas^7^. Therefore, to achieve optimal oral hygiene status**,** tooth brushing should be accompanied by the use of chemotherapeutic agents^8,9^.

Among the available antimicrobial agents, chlorhexidine gluconate (CHX) is considered effective for reducing oral biofilms, as well as for preventing and treating gingival inflammation^10^. However, CHX mouth-rinse has many side effects, including staining of the teeth, tongue, and restorations, as well as increased supragingival calculus formation and altered taste perception^8^. Therefore, researchers have been investigating natural bioactive compounds that may avoid these side effects. In particular, essential oils extracted from cinnamon, sweet basil, peppermint, and spearmint exhibit robust antibacterial activities^11^. Several studies have shown that cinnamon essential oil (CEO) inhibits the proliferation of *Streptococcus mutans* and *Porphyromonas gingivalis*, pathogenic bacteria that cause oral diseases, and the proliferation of *Candida albicans*, a pathogenic fungus implicated in the occurrence of denture-induced stomatitis^12,13^. Another study showed that CEO interfered with the formation of single-species biofilms formed using specific oral strains^11^. Although CEO reportedly exhibits antibacterial effects against a wide array of pathogenic oral bacteria, more information is needed regarding whether it can sufficiently suppress the formation of multi-species biofilms, which is essential for the prevention of oral diseases. Notably, oral diseases are caused by systematic and complex interactions among biofilms of more than 700 species of oral microorganisms, rather than by single species of bacteria in isolation^14,15^. More importantly, 80–90% of the matrixes in oral biofilms are composed of water^9^. Thus, to allow lipophilic CEO to penetrate into deep layers of oral biofilms, the oil must be emulsified with appropriate technology. However, there has been minimal research regarding the ability of CEO to inhibit multi-species biofilms present in the oral cavity.

Therefore, this study was performed to investigate whether cinnamon essential oil nanoemulsion (CEON) could inhibit the maturation of microcosm biofilms derived from saliva, by using quantitative light-induced fluorescence-digital (QLF-D), an optical device that visualizes pathogenic oral biofilms via red fluorescence. Furthermore, this study investigated the antibacterial effect of CEON against caries-causing aciduric bacteria in biofilms, as well as the growth-inhibiting effect of CEON against oral microorganisms in biofilms.

## Materials and methods

### Experimental design

This experimental in vitro study using bovine incisors was conducted from April 1, 2019 to December 30, 2019. Its protocol was approved by the Institutional Review Board of Gachon University, South Korea (approval no. 1044396-201904-HR-057-01). All experimental procedures were performed in full accordance with the Board’s relevant guidelines and regulations. In addition, saliva was collected from an adult volunteer following the acquisition of written informed consent, in accordance with the World Medical Association Declaration of Helsinki.

**Figure 1:**
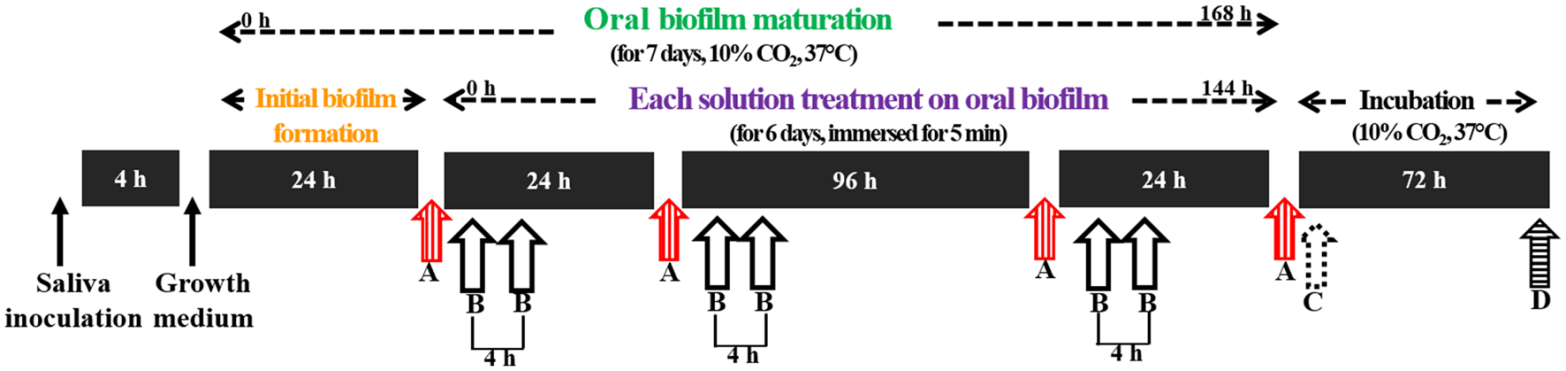
Flow chart of the experimental procedures. QLF-D quantitative light-induced fluorescence-digita, *CEON* cinnamon essential oil nanoemulsion, *CHX* chlorhexidine gluconate, *CB* cocamidopropyl betaine, *CFUs* colony-forming units. A: QLF-D analysis. B: Treatment of oral biofilms with 5% CEON, 0.12% CHX, or 0.5% CB. C: Harvest and absorbance measurement of oral biofilms. D: Assessment of aciduric bacteria CFUs.

An oral microcosm biofilm model using human saliva as an inoculum was employed, which allowed the maturation of biofilms on bovine incisors for 7 days. On the maturing biofilms, one of three solutions, 5% CEON, 0.12% CHX (positive control), or 0.5% cocamidopropyl betaine (CB, negative control), was applied twice per day for 6 days. To quantitatively analyze the inhibitory effects of the solutions on the formation of biofilms, specimens with biofilms were photographed using the QLF-D Biluminator (Inspektor Research Systems BV, Netherlands) once per day. QLF-D is a device that visualizes pathogenic oral biofilms via red fluorescence of porphyrin compounds, which are metabolites produced by oral bacteria species, using 405 nm visible blue light^16^. QLF-D has been recognized as a valuable tool for objectively and quantitatively evaluating the maturation of biofilms^16,17^. In addition, to evaluate growth inhibition of oral microorganisms in biofilms, absorbance was measured using the Multiskan FC Microplate Photometer (Thermo Fisher Scientific Inc., USA). Finally, to evaluate antibacterial effects against aciduric bacteria in biofilms, colony-forming units (CFUs) were counted. All analyses were performed by a single trained examiner. A brief summary of the experimental procedures is provided in Fig. 1.

### Preparation of enamel specimens

Enamel specimens were made using bovine incisors to generate oral biofilms. The 105 intact bovine incisors used in this study were collected from one slaughterhouse (Incheon, South Korea) following official approval by Incheon City. The mean age of the slaughtered animals was 3 years. Using the method described by Kim et al.^16^, the dental crowns of bovine incisors without cracks and white spots were separated from the corresponding roots, and their labial surfaces were sectioned with a low-speed saw using a diamond-coated disc (6 mm × 5 mm). The specimens were then embedded in a circular acrylic mold using a dental impression material. The exposed enamel surfaces were ground flat with a water-cooled polishing unit (M-Prep 5; Allied High Tech Products Inc., USA) using 360-grit abrasive sandpaper for 20 s, 600-grit paper for 20 s, and 1200-grit paper for 10 s (SiC Sand Paper; R&B Inc., South Korea) operating at 100 rpm. Finally, to secure space for the accumulation of oral biofilms, the specimen was re-embedded 1 mm below the peak of the acrylic mold. The minimum number of specimens required for one-way analysis of variance was determined to be 102, based on a power calculation done using G^*^Power 3.1 (Franz Faul, Germany) with the following parameters: 95% power, 5% significance level, and 0.4 effect size^18^. The specimens were randomly assigned to one of the following three groups: 5% CEON (n = 35), 0.12 CHX (n = 35), or 0.5% CB (n = 35).

### Preparation of CEON

An essential oil extracted from cinnamon (*Cinnamomum verum*) bark by steam distillation was purchased from dōTERRA International, LLC (USA). Steam distillation is a technique that can extract many components inhibiting the growth of microorganisms, and has been widely used in various studies^19,20^. The chemical constituents of this CEO were analyzed using gas chromatography-mass spectrometry (7890A/5975C; Agilent Technologies, USA). An HP-5MS fused-silica capillary column (30 m × 0.25 mm × 0.25 μm) was used. The operating conditions were as follows: initial oven temperature, 40 °C for 5 min, 10 °C for 5 min, and 280 °C for 5 min; inlet temperature, 250 °C; carrier gas, 1.0 mL/min helium; injection volume, 1 μL; split ratio, 10:1; electron ionization voltage, 70 eV; mass spectrometer ion source temperature, 230 °C; and mass spectrometer scan range, 30–500 m/z. All components of the CEO were identified by comparing their mass spectra with the standard mass spectra provided by the NIST 11 library. The results showed that cinnamaldehyde constituted > 50% of the total peak area (61.80%). Fourteen other compounds were identified, including acetic acid cinnamyl ester (7.81%), caryophyllene (6.89%), β-thujene (5.70%), 3-allyl-6-methoxyphenol (4.35%), o-cymene (2.77%), and α-phellandrene (2.06%) (seven compounds of less than 2% are not shown). To determine the concentration of CEO used for treatment of biofilms, a disc diffusion assay was performed to measure its antibacterial activity against oral microorganisms present in saliva, because saliva contains most of the bacteria in oral biofilms^21^. Furthermore, several preliminary studies were performed, including minimal biofilm inhibitory concentration analysis, using the Multiskan FC Microplate Photometer (Thermo Fisher Scientific Inc.). Based on the analysis results, the concentration of CEO used in this study was set at 5%. In addition, to improve delivery of the CEO active ingredients, the oil was formulated in an oil-in-water type emulsion with nano-sized oil droplets, on the basis of several previous studies^22,23^. CB (Combi-Blocks Inc., USA), a biosurfactant, and an ultrasonicator (VCX 750; Sonics & Materials Inc., USA) were used for emulsification. Initially, stock CEO (5 mL) was dissolved in 0.5% CB (0.5 mL). It was then diluted in sterile distilled water (94.5 mL) to prepare a 5% CEO emulsion. To minimize the size of the oil droplets, the emulsion was subjected to high-intensity ultrasonication for 5 min using an ultrasonicator (VCX 750; Sonics & Materials Inc.), as follows: pulse, start for 2 s and stop for 3 s; frequency, 20 kHz; amplitude, 20%. Finally, a vortex mixer (VM-96A; Lab Companion, South Korea) was operated at 3000 rpm for 5 min to remix the CEON immediately before addition to biofilms.

The mean size of the oil droplets in the formulated emulsion was analyzed using a zeta potential and particle size analyzer (ELSZ-2000ZS; Otsuka Electronics, Japan). The size was measured three times in succession at 25 °C by means of dynamic light scattering, which is the most commonly used technique for nanoparticle sizing. The oil droplet size was 206.2 nm in the first round, 208.2 nm in the second round, and 207.1 nm in the third round. The mean droplet size was 207.2 nm (data not shown).

### Formation of microcosm oral biofilms and treatment with three solutions

To induce the growth of multi-species pathogenic biofilms, the biofilms were formed on enamel specimens for 7 days under a basal medium mucin with 0.5% sucrose, based on an established oral microcosm biofilm model^16,24–26^. The basal medium of synthetic saliva contained 2.5 g/L porcine mucin (Type III; Sigma-Aldrich, USA), 10.0 g/L proteose peptone (KisanBio, South Korea), 5.0 g/L trypticase peptone (KisanBio), 5.0 g/L yeast extract (KisanBio), 1 mmol/L urea (GeorgiaChem, USA), 1 mmol/L arginine (GeorgiaChem), 2.5 g/L potassium chloride (OCI Co., South Korea), 5 mg/L hemin (Sigma-Aldrich, USA), and 1 mg/L menadione (Sigma-Aldrich). This medium was adjusted to pH 7.0 using a 50% sodium hydroxide solution (Daejung Chemicals & Metals Co., South Korea). On the maturing biofilms, one of the three treatment solutions, i.e., 5% CEON, 0.12% CHX (Unimed Pharmaceuticals Inc., South Korea) or 0.5% CB (Combi-Blocks Inc.), was applied twice per day at 4-h intervals for 6 days. First, whole stimulated saliva to be used as the inoculum was collected from a healthy woman who did not have any active dental caries or periodontal diseases, and had not taken antibiotics in the preceding 3 months. The donor was instructed not to perform any oral hygiene practices for 24 h prior to saliva collection. The stimulated saliva was collected using paraffin wax (Ivoclar Vivadent, Germany) and then filtered through sterilized glass wool (Duksan Pure Chemical, South Korea). The prepared saliva (1.5 mL) was immediately used to inoculate enamel specimens in a 24-well cell culture plate, and the plate was incubated anaerobically at 37 °C for 4 h. Next, the saliva was aspirated from the well, and 1.5 mL growth medium was added (mixture of 0.1 mL of 0.5% sucrose and 1.4 mL of basal medium mucin). The plate was again incubated at 37 ℃ with 10% CO_2_ for 24 h. After 24 h, the specimens on which biofilms had initially formed were immersed in 1.5 mL of one of the three treatment solutions in each well of a 24-well plate for 5 min. Next, the specimens treated with biofilms were gently washed with 1.5 mL sterile distilled water in a new well for 5 s, and then carefully placed into a new well in a 24-well plate containing new basal medium mucin (1.5 mL). The plate was incubated again under the same culture conditions. After 4 h, a second treatment was performed on each specimen with biofilms in the manner described above. All treatment procedures were repeated for 6 days.

### Analysis of antibacterial and antibiofilm activities of the three treatment solutions

#### Inhibitory effect against formation of oral biofilms

To evaluate the inhibitory effect against formation of oral biofilms, QLF-D was used in this study, as in a prior study^16,17,24^. Biofilms were photographed once per day, immediately prior to treatment with the designated solution. QLF-D images were taken under the following conditions, while maintaining a consistent distance between the camera lens and each specimen of biofilm: shutter speed, 1/60 s; aperture value, 5.0; and ISO speed, 1600^27^. For fluorescence images, red and green values in the same area on each biofilm specimen were analyzed. The mean red/green ratio (R/G values) was then calculated using the ImageJ image analysis program (version 1.46; NIH, USA). Higher R/G values were presumed to indicate advanced maturity and greater pathogenicity^16,27^.

#### Antimicrobial effect against aciduric bacteria in biofilms

Colony-forming units (CFUs/mL) were counted to evaluate the antibacterial effect against aciduric bacteria in biofilms^28^. Briefly, 24 h after biofilms had received the final treatment with the designated solution, the specimens with biofilms were rinsed with sterile distilled water (1.5 mL) to remove the remaining growth medium. Then, specimens were transferred into conical tubes containing 2 mL of distilled water, and the attached biofilms were dispersed using a sonicator (SHB-1025; Saehan Sonic, South Korea) and a vortex mixer (VM-96A; Jeio Tech, South Korea) (1 min each). The prepared bacterial suspensions were serially diluted (10^−1^–10^−6^) and spread on brain heart infusion agar plates adjusted to pH 4.8. The plates were incubated anaerobically at 37 °C with 10% CO_2_ for 72 h, and the number of aciduric bacterial CFUs was counted by a single examiner. A higher number of CFUs was presumed to indicate that aciduric bacteria were more prevalent in biofilms^16^.

#### Inhibitory effect against oral microorganism growth in biofilms

Absorbance was measured using the Multiskan FC Microplate Photometer (Thermo Fisher Scientific Inc.), to quantify the inhibitory effect against oral microorganism growth in biofilms. Each diluted bacterial suspension (100 µL), identical to the suspension used for analyzing the antimicrobial effect against aciduric bacteria, was transferred into a new 96-well plate and its absorbance was then measured at a wavelength of 595 nm using the method described by Jeong et al.^29^. A lower absorbance was presumed to indicate greater inhibition of oral microorganism growth.

### Statistical analysis

For statistical analysis, IBM SPSS Statistics (ver. 23.0; IBM Corp., USA) was used, and *p* < 0.05 was considered to indicate statistical significance. Regarding the R/G values of biofilms, two-way repeated-measures analysis of variance, followed by the Bonferroni post hoc test, was performed to verify the interaction effects between time and each treatment group. To compare the number of aciduric bacterial CFUs in biofilms and the absorbance of bacterial suspensions among the three treatment solutions, one-way analysis of variance and Scheffe’s post hoc test were performed.

## Results

### R/G values of 7-day-old mature biofilms according to treatment solution

**Figure 2:**
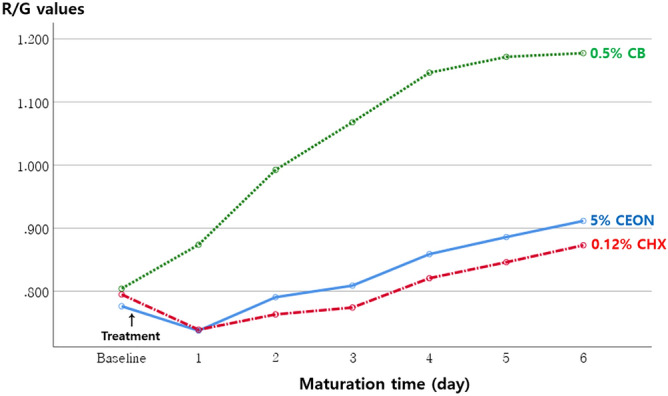
Changes in R/G values of oral biofilms formed in the presence of three treatment solutions according to maturation time. *CEON* cinnamon essential oil nanoemulsion, *CHX* chlorhexidine gluconate, *CB* cocamidopropyl betaine.

**Figure 3:**
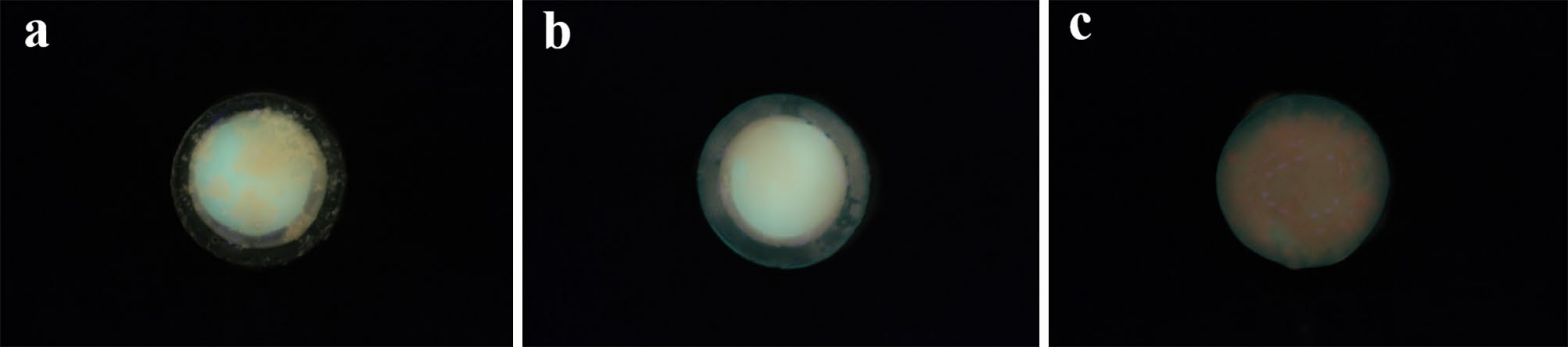
QLF-D red fluorescence images of 7-day-old mature oral biofilms after treatment with 5% CEON (**a**), 0.12% CHX (**b**), and 0.5% CB (**c**). *CEON* cinnamon essential oil nanoemulsion, *CHX* chlorhexidine gluconate, *CB* cocamidopropyl betaine.

The R/G values of 7-day-old mature biofilms exposed to each of the treatment solutions are shown in Table 1. The R/G values of initial biofilms before applying the treatment solutions to the specimens did not differ significantly among the three groups (*p* > 0.05). After applying the treatment solutions, the R/G values of biofilms differed significantly between the 5% CEON and 0.5% CB groups at all maturation time points, but not between the 5% CEON and 0.12% CHX groups (*p* > 0.05, Fig. 2). The R/G values of 7-day-old mature biofilms were lowest in the 0.12% CHX group (0.87 ± 0.09), followed by the 5% CEON group (0.91 ± 0.10) and the 0.5% CB group (1.18 ± 0.07) (*p* < 0.001). There was no significant difference in R/G values between the 5% CEON and 0.12% CHX groups (*p* > 0.05). Representative QLF-D red fluorescence images of 7-day-old mature biofilms for each treatment solution are shown in Fig. 3.

**Table 1:** R/G values of 7-day-old mature biofilms according to treatment solution.

Groups	Treatment solutions	N	R/G values			
			Baseline	F(*p*)	7-day-old mature biofilms	F(*p*)
Experimental	5% CEON	35	0.77 ± 0.05	1.951 (0.147)	0.91 ± 0.10^a^	111.281 (< 0.001)
Positive control	0.12% CHX	35	0.79 ± 0.05		0.87 ± 0.09^a^	
Negative control	0.5% CB	35	0.79 ± 0.06		1.18 ± 0.07^b^	

*CEON* cinnamon essential oil nanoemulsion, *CHX* chlorhexidine gluconate, *CB* cocamidopropyl betaine.

*p* values obtained from one-way analysis of variance. All values are expressed as means ± standard deviations.

^a,^^b^Different letters indicate significance by Bonferroni multiple comparison test at α = 0.05.

### Aciduric bacterial CFUs within biofilms according to treatment solution

The aciduric bacterial CFUs within biofilms exposed to each treatment solution are shown in Table 2. The number of aciduric bacterial CFUs was lowest in the 5% CEON group (4.81 ± 2.03), but this did not significantly differ from the number in the 0.12% CHX group (5.11 ± 1.65, *p* > 0.05). The number of CFUs was highest in the 0.5% CB group (6.93 ± 0.31, *p* < 0.001).

**Table 2:** Numbers of aciduric bacterial CFUs within biofilms according to treatment solution.

Groups	Treatment solutions	N	Log_10_ CFUs/mL	F	*p*
Experimental	5% CEON	35	4.81 ± 2.03^a^	19.823	< 0.001
Positive control	0.12% CHX	35	5.11 ± 1.65^a^		
Negative control	0.5% CB	35	6.93 ± 0.31^b^		

*CFUs* colony-forming units, *CEON* cinnamon essential oil nanoemulsion, *CHX* chlorhexidine gluconate, *CB* cocamidopropyl betaine.

*p* value obtained from one-way analysis of variance. All values are presented as means ± standard deviations.

^a,^^b^Different letters indicate significance by Scheffe’s multiple comparison test at α = 0.05.

### Absorbances of bacterial suspensions according to treatment solution

The absorbances of bacterial suspensions exposed to each of the treatment solutions are shown in Table 3. The absorbance was lowest in the 5% CEON group (0.29 ± 0.16) and highest in the 0.5% CB group (1.14 ± 0.17, *p* < 0.001). The absorbance of the 5% CEON group was not significantly different from that of the 0.12% CHX group (0.36 ± 0.10, *p* > 0.05).

**Table 3:** Absorbance of oral bacterial suspensions according to treatment solution.

Groups	Treatment solutions	N	Absorbance	F	*p*
Experimental	5% CEON	35	0.29 ± 0.16^a^	346.725	< 0.001
Positive control	0.12% CHX	35	0.36 ± 0.10^a^		
Negative control	0.5% CB	35	1.14 ± 0.17^b^		

*CEON* cinnamon essential oil nanoemulsion, *CHX* chlorhexidine gluconate, *CB* cocamidopropyl betaine.

*p* value obtained from one-way analysis of variance. All values are expressed as means ± standard deviations.

^a,^^b^Different letters indicate significance by Scheffe’s multiple comparison test at α = 0.05.

## Discussion

This study evaluated the antibacterial and antibiofilm activities of 5% CEON against multi-species biofilms, which are the root cause of oral disease. In general, to achieve a robust antibacterial effect from CEO, the oil must be pre-treated prior to application, considering the nature of the target, due to the lipophilic characteristics of the CEO^30^. The oral biofilms were mostly composed of water^9^, and the oral microorganisms present in the biofilms were embedded within a self-produced matrix of extracellular polymeric compounds^3,9,31^. Although cinnamaldehyde (a major constituent of CEO that substantially contributes to its antibacterial properties) is biocompatible and low in toxicity^32,33^, oral adverse reactions have been reported sporadically, such as intraoral allergic reactions and contact stomatitis when used at high concentrations^34,35^. Accordingly, the concentration of CEO in a treatment solution should be as low as possible to minimize possible side effects from the components of cinnamon. Therefore, considering these characteristics of oral biofilms and CEO, we used CB, an organic surfactant with low toxicity^36^, distilled water, and high-intensity ultrasonic waves to produce an oil-in-water type of CEON. Ultrasonication is a technique that contributes to the conversion from a coarse emulsion to a nano-sized emulsion by reducing the oil droplet size^37^. We applied this 5% CEON, containing oil droplets with a mean size of approximately 200 nm, onto maturing biofilms twice per day for 6 days, and then compared the effects of 5% CEON with those of two other treatment solutions. The results showed that the red fluorescence intensity of 7-day-old mature biofilms was lowest in the 0.12% CHX group, which was not significantly different from the intensity in the 5% CEON group. In addition, the R/G values of biofilms did not significantly differ between the 5% CEON and 0.12% CHX groups at all maturation time points, whereas significant differences were observed between the 5% CEON and 0.5% CB groups (Fig. 2). Thus, 5% CEON was similar to 0.12% CHX in terms of its ability to significantly inhibit the maturation of both early biofilms and mature biofilms, even when the biofilms were incubated in an optimal environment for enhancement of pathogenicity. Furthermore, in the 5% CEON group, the growth of oral microorganisms, including caries-causing aciduric bacteria, was suppressed the most. We confirmed that this effect was not significantly different from that of the CHX solution, the current gold-standard antimicrobial agent.

We could not directly compare the results of this study with those of previous studies, because we were unable to identify any publications that analyzed the effects of CEO on multi-species oral biofilms. However, our findings are consistent with the results of a study in which a 24-h pre-established *Streptococcus mutans* biofilm was exposed to CEO^11^. In that study, the biofilm mass decreased by > 50%, although the oil had been applied to a single-species oral biofilm^11^, in contrast to the present study. We presumed that our nanoemulsion technique (using an ultrasonicator and biosurfactant) contributed to deeper penetration of CEO active ingredients into mature biofilms, thus resulting in significant antibacterial and antibiofilm activities, despite the low CEO concentration. Research on the nanoemulsification of CEO, aiming to enhance its antibacterial activity against dental biofilms, is limited in the dental field. However, compared with conventional emulsions, the smaller droplet diameters of nanoemulsions reportedly facilitate stronger antibacterial properties against gram-positive and gram-negative bacteria^38,39^. In addition, the inhibitory effects of 5% CEON on the growth of oral microorganisms and the formation of pathogenic multi-species oral biofilms, which were demonstrated in the present study, may have been mediated by various active substances within cinnamon (e.g., cinnamaldehyde)^12,19^. Although the mechanism underlying the antibacterial effect of CEO has not yet been fully elucidated, these active constituents have been reported to cause cell lysis by cell membrane distortion^37^. In particular, cinnamaldehyde is highly electronegative^40^. Electronegative compounds interfere with biological processes (e.g., electron transfer), react with nitrogen-containing components such as proteins and nucleic acids, and ultimately inhibit microorganism growth^41^. In an in vitro study of cariogenic bacteria, aqueous cinnamon extract effectively suppressed acid production by *Streptococcus mutans*, as well as its bacterial adhesion^42^. In a study by Zainal-Abidin et al.^12^, CEO inhibited the proliferation of *Porphyromonas gingivalis* and *Fusobacterium nucleatum*, two periodontopathic bacteria, by inducing changes in their surface membranes. Wang et al.^20^ observed that higher concentrations of CEO or cinnamaldehyde led to greater leakage of proteins and nucleic acids. Therefore, on the basis of our findings and the results of previous studies, we recommend consideration of CEO as a potential natural antimicrobial agent that can aid in controlling oral diseases by effectively inhibiting the formation of pathogenic oral biofilms.

To the best of our knowledge, this study is the first to systematically demonstrate that CEON significantly inhibits the maturation of pathogenic multi-species biofilms, which were formed using a microcosm biofilm model. Notably, our study used a QLF-D device that can quantitatively evaluate the maturity of biofilms^16^ and non-destructively monitor the effects of antimicrobial agents against those biofilms^27^.

However, there were some limitations. First, because this was an in vitro study, the antibacterial and antibiofilm activities of 5% CEON may not reflect those in the actual oral environment, wherein they would interact with saliva. Second, because the treatment period was relatively short, the study only assessed the short-term effect of 5% CEON. Finally, the properties of biofilms formed on bovine incisors may differ from those of oral biofilms that accumulate on human teeth. Therefore, in future studies, the antibiofilm activity of CEO should be assessed according to both concentration and long-term effectiveness, in a larger number of enamel specimens. Furthermore, understanding the mechanism by which CEON inhibits the maturation of multi-species biofilms could enhance the usefulness of CEO in the prevention and reduction of oral diseases caused by biofilms. Finally, although cinnamon-related contact stomatitis is a relatively uncommon disorder^34,43^, CEON-related hypersensitivity reactions involving the oral mucosa should be evaluated in the future.
